# Update on the Effect of the Urinary Microbiome on Urolithiasis

**DOI:** 10.3390/diagnostics13050951

**Published:** 2023-03-02

**Authors:** Hae Do Jung, Seok Cho, Joo Yong Lee

**Affiliations:** 1 Department of Urology, Inje University Ilsan Paik Hospital, Inje University College of Medicine, Goyang 10380, Republic of Korea; 2 Department of Urology, Severance Hospital, Urological Science Institute, Yonsei University College of Medicine, Seoul 03722, Republic of Korea; 3 Center of Evidence Based Medicine, Institute of Convergence Science, Yonsei University, Seoul 03722, Republic of Korea

**Keywords:** microbiota, urolithiasis, urinary tract

## Abstract

Microbiota are ecological communities of commensal, symbiotic, and pathogenic microorganisms. The microbiome could be involved in kidney stone formation through hyperoxaluria and calcium oxalate supersaturation, biofilm formation and aggregation, and urothelial injury. Bacteria bind to calcium oxalate crystals, which causes pyelonephritis and leads to changes in nephrons to form Randall’s plaque. The urinary tract microbiome, but not the gut microbiome, can be distinguished between cohorts with urinary stone disease (USD) and those without a history of the disease. In the urine microbiome, the role is known of urease-producing bacteria (*Proteus mirabilis*, *Klebsiella pneumoniae*, *Staphylococcus aureus*, *Pseudomonas aeruginosa*, *Providencia stuartii*, *Serratia marcescens*, and *Morganella morganii*) in stone formation. Calcium oxalate crystals were generated in the presence of two uropathogenic bacteria (*Escherichia coli* and *K. pneumoniae)*. Non-uropathogenic bacteria (*S. aureus* and *Streptococcus pneumoniae*) exhibit calcium oxalate lithogenic effects. The taxa *Lactobacilli* and *Enterobacteriaceae* best distinguished the healthy cohort from the USD cohort, respectively. Standardization is needed in urine microbiome research for urolithiasis. Inadequate standardization and design of urinary microbiome research on urolithiasis have hampered the generalizability of results and diminished their impact on clinical practice.

## 1. Introduction

Microbiomes are defined as microbiota, their genomes, and the surrounding environmental conditions. Microbiota are ecological communities of commensal, symbiotic, and pathogenic microorganisms [1,2]. Sometimes, the term “microbiome” is used interchangeably with “microbiota.” Microorganisms are conventionally classified into pathogens, normal flora, and probiotics; however, normal flora have been recently referred to as “indigenous microbiota”.

Research on the human urinary microbiome has the potential to increase understanding of a variety of urologic disorders, including lower urinary tract symptoms (LUTS) and urologic cancer [3,4,5]. Many recent studies have reported the role of the microbiome in urinary stone formation (urolithiasis) [6,7,8,9,10,11,12,13,14,15]. Urolithiasis can be classified as being caused by infection (magnesium ammonium phosphate, carbonate apatite, and ammonium urate), non-infectious causes (calcium oxalate, calcium phosphate, and uric acid), or hereditary abnormalities (cystine, xanthine, and 2,8-Dihydroxyadenine) [16].

Urolithiasis is a disease with a high recurrence rate, and its occurrence rate is rising worldwide [17]. As a direct consequence of urolithiasis, patients with stone recurrence have a deterioration in their quality of daily life, and the financial burden associated with managing urolithiasis is increasing [18]. Thus, the various etiologies, including the microbiome of urolithiasis, which can be a potential cause for stone recurrence, must be understood. 

Here, we provide a reviewing update focusing on the role of the urinary microbiome in urolithiasis.

## 2. Pathophysiology of Urinary Stone Formation

**Figure 1:**
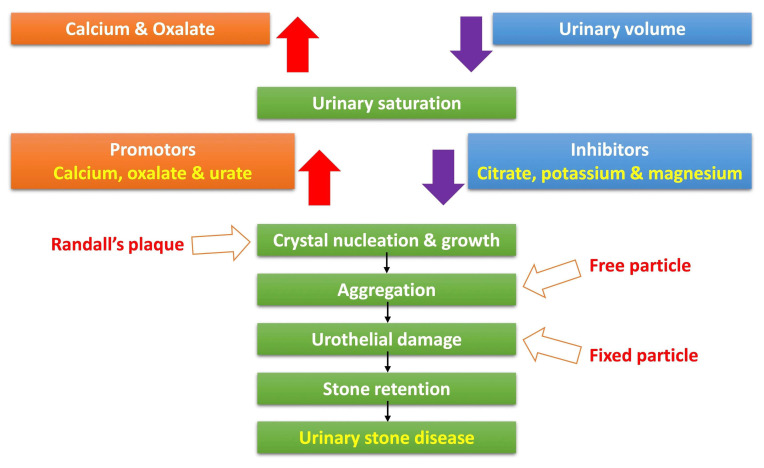
Process of calcium oxalate stone formation.

Figure 1 shows the process of calcium oxalate stone formation. An increase in calcium and oxalate and a decrease in urine volume initiate urinary stone formation and urine saturation. Among these processes, the formation of Randall’s plaque is related to crystal nucleation and growth. The formation of free particles is related to crystal aggregation. The formation of fixed particles is related to urothelial damage, and through this process, stone retention is repeated and develops into urinary stone disease (USD). The urinary microbiome could be involved in stone formation through hyperoxaluria and calcium oxalate supersaturation, biofilm formation and aggregation, and urothelial injury.

## 3. History of the Urinary Microbiome Studies

The Human Microbiome Project (HMP) aimed to explore microbial communities and their connections to their human hosts [19,20]. The HMP was launched in 2008. Initially, however, the HMP did not include the bladder [9]. Samples were obtained from the lungs, skin, oral cavity, gastrointestinal tract, and vagina. Urine was once believed to be sterile in healthy persons, despite including several microorganisms.

Modern clinicians have linked bacteria in the urine to infection or, less frequently, an undefined syndrome called “asymptomatic bacteriuria” [21]. The “sterile urine” paradigm has been the foundation for this and other existing ideas for a long time [21]. However, microbial communities (microbiota) have recently been found in the female urinary bladder [22,23,24,25,26,27,28,29,30]. As a result, the concept of “sterile urine” is obsolete.

In response, new methods to study urine have been developed. Urine initially found to have “no growth” by the usual methodology has been shown to include bacteria that could be cultivated using 16S rRNA sequence analysis [22]. As a result, Hilt et al. [28] have developed an enhanced quantitative urine culture (EQUC) methodology that employs 100 times as much urine as a traditional culture and a wide range of media and environmental conditions to isolate and identify many organisms missed by conventional culturing. When comparing EQUC to the conventional clinical technique, a 90% false negative rate was found for the conventional clinical approach [28].

The previous widespread testing methods (such as the standard urine culture and dipstick) are inadequate for research purposes and may also be inappropriate for clinical purposes. However, the clinical feasibility and future roles of 16S rRNA sequencing and EQUC have yet to be determined [21]. 

In addition, knowledge of the “normal” urinary microbiome has been hindered because there was no formal definition of bladder health [31]. The Prevention of Lower Urinary Tract Symptoms Consortium has recently defined bladder health as “A complete state of physical, mental, and social well-being related to bladder function and not merely the absence of LUTS” [32]. 

However, although it is difficult to identify a single group of common bacteria found in a healthy bladder, comparative studies have provided insights into the overlap and various compositions of urine-based microbial communities [33]. One study found that the urinary microbiome is composed primarily of species from a few genera, most frequently *Lactobacilli* (the generic term *‘Lactobacilli’* is useful to designate all organisms that were classified as *Lactobacillaceae* [34]), *Gardnerella*, and *Streptococcus*, and has a lower biomass than the vaginal microbiome [31]. 

Due to the significant anatomical and physiological differences between men’s and women’s lower urinary tracts, it is not surprising that the urine microbiome of the bladder also exhibits unequal stratification [33]. One study discovered that *Firmicutes*, *Actinobacteria*, *Bacteroidetes*, and *Proteobacteria* were the phyla that men and women shared most frequently. However, in general, in healthy females, *Streptococcus*, *Lactobacilli*, and *Prevotella* are abundant, whereas, in males, *Lactobacilli*, *Corynebacterium*, and *Gardnerella* are more prevalent [35]. Another study found that *Lactobacilli* predominates in the bladder in healthy women, while the bladders of healthy men contain high levels of *Enterococcus*, *Proteus*, and *Klebsiella* in their bladder [29].

Urine samples obtained by catheterization and 16S rRNA sequencing were used in a multicenter cross-sectional study by the NIH-NICHD-funded Pelvic Floor Disorders Network [36]. They described characteristics of the urine microbiomes of well-characterized asymptomatic women (*n* = 84) compared to those of women with mixed urinary incontinence (MUI) (*n* = 123). These researchers confirmed earlier reports that some members of the genus *Lactobacilli* may be linked to urinary symptoms, including urgency urinary incontinence, even though the proportion of women with predominant *Lactobacilli* did not differ between women with MUI and asymptomatic women with matching ages [36].

Bajic et al. [37] classified 28 men with surgical histories of benign prostate enlargement (BPE)–LUTS and 21 men with histories of non-BPE–LUTS surgery using the International Prostate Symptom Score (IPSS). The authors obtained and analyzed paired catheterized and voided urine samples using EQUC and 16S rRNA sequencing. Overall, 39% of the urine specimens obtained by catheterization and 98% of specimens obtained by self-voiding included microbiome, and there was a significant difference between the microbiome of these types of urine specimens. The findings of the study suggest that catheterized urine may be a better method for collecting the urinary microbiome of male patients compared to voided urine. They examined catheterized urine samples from a group of men, some with and some without BPE, and discovered that the severity of LUTS was linked to the presence of bacteria in bladder urine. Specifically, the study showed that men with mild, moderate, and severe LUTS had detectable bacteria in their bladder urine at rates of 22.2%, 30.0%, and 57.1%, respectively (*p* = 0.024). The authors determined that severe LUTS in men may be related to detectable bladder bacteria compared to men with milder or no symptoms.

Few studies have examined the relevance of the urinary microbiome in urological cancers. A study compared the urine of six healthy people with that of eight bladder cancer patients and measured an increased amount of the genus *Streptococcus* in the bladder cancer group using 16S rRNA sequencing [38]. A more recent study compared urine samples using 16S sequencing from twelve patients diagnosed with bladder cancer and eleven healthy participants selected from an age-matched cohort [39]. Although there were no significant differences between the two groups in the diversity or overall composition of the microbiome, certain taxa were considerably over-represented in either the bladder cancer or healthy subgroups. The most prevalent bacteria in the healthy subgroup were *Veillonella*, *Streptococcus*, and *Corynebacterium*. In contrast, numerous taxa were highly represented in the bladder cancer group, including members of the colorectal cancer-associated genus *Fusobacterium*.

## 4. Urinary Microbiome and Urolithiasis

In the urine microbiome, the role of urease-producing bacteria in stone formation is already understood. Urease-producing bacteria are *Proteus mirabilis*, *Klebsiella pneumoniae*, *Staphylococcus aureus*, *Pseudomonas aeruginosa*, *Providencia stuartii*, and *Serratia marcescens* and *Morganella morganii*. Those bacteria break down urea and induce ammonia and carbon dioxide production, leading to renal tubular injury, urine alkalinization, and subsequent formation of phosphate salts that urease-producing bacteria form struvite stones [8]. 

Recent studies have shown that enterobacteria in the urinary microbiome, including *Escherichia coli*, may be associated with urolithiasis. In a study to determine if the presence of uropathogenic *E. coli* affected calcium deposition in the urinary tracts of mice, calcium deposition was 2.7-fold higher after inoculation with *E. coli* [40]. 

According to Hirano et al. [41], the aggregation of crystalline and organic matter in the urine of stone formers may be due to in part to the adhesive properties of certain bacteria. These bacteria have the ability to actively participate in the formation of stones by causing the aggregation of these materials. *E. coli* and *P. mirabilis* worsened calcium oxalate encrustation on the surface of polyurethane film, the substance used in urinary stents, as demonstrated by Venkatesan et al. [42]. The authors further proposed that the biofilm produced by these bacteria might cause calcium oxalate encrustation. Bacteria bind to calcium oxalate crystals, causing pyelonephritis, which leads to changes in nephrons that form Randall’s plaque. In addition, *E. coli* decreases citrate levels by secreting citrate lyase, which causes calcium oxalate supersaturation [40]. Ultimately, bacteria attach to the urothelium and repeat this process.

Chutipongtanate et al. [43] examined the lithogenic potential of Gram-negative and Gram-positive bacteria on calcium oxalate. The researchers employed both morphological evaluation and a new screening method, as well as gold-standard assays, to determine that bacteria have the capacity to directly enhance the growth and aggregation of calcium oxalate crystals. This study was the first to utilize this novel screening method. In this study, calcium oxalate crystals were generated in the presence of two uropathogenic bacteria, i.e., *E. coli* and *K. pneumoniae*. In addition, the non-uropathogenic bacteria, i.e., *S. aureus* and *S. pneumoniae*, exhibited calcium oxalate lithogenic effects.

Because not all urinary tract infection patients develop calcium oxalate stones, it can be understood as a hypothesis, but the microbes in the urine are not entirely essential for forming calcium oxalate stones. Even then, we must consider that the role of the urinary microbiome for stone formation in chronic kidney disease and kidney transplant patients could be more critical due to the perturbation (dysbiosis) of the urinary microbiome induced by immunosuppressive medications and repeated antibiotic treatments [44,45].

Recently, Kachroo et al. [12] have performed the first comparative shotgun metagenomic analysis of the urinary microbiome from patients either with pure calcium oxalate stones with an active episode of USD or without a history of USD. The goal of this study was to compare the shotgun metagenomics of voided midstream urine samples from a small number of patients (*n* = 5 calcium oxalate stone formers, *n* = 5 healthy controls) to identify the associated microbial functions across prokaryotic, viral, fungal, and protozoan domains. According to their findings, the genes involved in oxalate metabolism, transmembrane transport, proteolysis, and oxidation-reduction pathways were expressed at lower levels in calcium oxalate stone formers. Genes enriched in the control group mapped overwhelmingly to *Lactobacillus crispatus* from 17 draft genomes taken from the data and more than 42,000 full-length reference genomes, while genes related to calcium oxalate mapped to *P. aeruginosa* and *Burkholderia sp*. 

A recent comparative multi-omics clinical study by Zampini et al. revealed that the urinary microbiome, but not the gut microbiome, could be distinguished between cohorts with USD (excluding cases of infectious stones) and those without a history of the disease [46]. In the urine microbiome, 8.8% of the operational taxonomic units (OTUs) were differently abundant, with 1.6 times more OTUs in the healthy group than in the USD group. *Lachnospiraceae* in the stool of the USD cohort, *Lactobacilli* in the urine of the healthy cohort, and *Enterobacteriaceae* in the urine of the USD cohort were the taxa that best distinguished the healthy cohort from the USD cohort. When assessing the differential concentrations of specific metabolites by USD status, 53 were enriched in the healthy group and 16 were enriched in the USD group, a 3.3-fold greater number of enriched metabolites in the healthy cohort compared to the USD cohort. This is the first study to use metagenomics to compare the urinary microbiome in USD and healthy persons. Multiple lines of evidence in this study indicate that the urinary microbiome contributes more to the development of USD than the gut microbiome. The microorganisms in urinary microbiome associated with urolithiasis is summarized in Table 1.

## 5. Standardization of Urinary Microbiome Research for Urolithiasis

Since the discovery of the human urinary microbiome, various technological and participant-related issues and irregular sampling settings have impacted urinary microbiome research [31]. In other words, the inadequate standardization and design of urinary microbiome research on urolithiasis have hampered the generalizability of results and diminished their impact on clinical practice [11]. Thus, in the United States, standardization of microbiome studies for urolithiasis has begun with the MICRObiome contributions on the Complexity Of the Stone Matrix (MICROCOSM)—the first international consortium focused on microbiome-urolithiasis research [13]. MICROCOSM was created to reduce the inconsistencies in microbiome research findings brought on by experimental factors, including sample collection, storage, DNA extraction, sequencing, and data analysis. 

In addition, taxonomic assignment using OTUs or amplicon sequence variants (ASVs) for the sequencing data could address the variation in results due to differences in the experimental design or population characteristics [14]. With OTUs’ classification, in the urinary tract, the family *Enterobacteriaceae* and the genus *Veillonella* were most related with USD patients, whereas the genus *Lactobacilli* was most connected with healthy people. However, with ASVs assignment, *Veillonellaceae* were more frequently found in the urine microbiome of healthy participants, whereas *Actinomycetaceae* and *Enterobacteriaceae* were most frequently found in USD patients [14]. 

Brubaker et al. [5] have proposed guidelines developed at the UROBIOME 2020 conference for documenting and reporting data originating from urinary microbiome studies. Key recommendations for urinary microbiome research are: first, using appropriate nomenclature to describe the urine specimen: “Urogenital” sample for voided urine samples versus “Urinary Bladder” for catheterized (transurethral or suprapubic aspirates). Second, aligning the urine sampling technique with the purpose of the research. For studies of bladder urinary microbiome in adult women, the authors recommend the collection of catheterized urine samples when possible. Third, using nucleic acid preservatives and maintaining cold temperatures while expediting sample transfer to the laboratory. Fourth, aligning the sample processing plan with the research question (culture±culture-independent techniques). Fifth, minimizing the batch effects as much as possible (differences in kits and sequencing runs; record information to account for differences in downstream analyses if necessary). Finally, adopting a standard metadata checklist for urinary microbiome studies.

## 6. Gut Microbiome and Urolithiasis

The gut and kidney appear to have a bidirectional relationship, as supported by growing evidence [47]. Research suggests that the gut microbiome plays a significant role in the gut–kidney axis [48], and disruption of the gut microbial community, or dysbiosis, has been linked to the development of several renal disorders. This may indirectly contribute to chronic kidney disease and hypertension [49], as well as urinary stone disease [50]. 

Before current sequencing techniques were developed, it had already been demonstrated that the gut microbiome forms stones. That is, the absence of *Oxalobacter formigenes* leads to the formation of stones [51]. Bacteria that use oxalate as an energy source are called oxalotrophs. *Lactobacilli* and *Bifidobacterium* are examples of generalist oxalotrophs, which are able to degrade substances beyond oxalate for carbon energy. On the other hand, *Oxalobacter formigenes* is an example of a specialist oxalotrophs. This bacterium can degrade oxalate in the intestinal tract via the expression of two enzymes, Formyl-CoA transferase and Oxalyl-CoA decarboxylase [8].

Siener et al. [52] presented clinical results that the absence of *Oxalobacter formigenes* induces hyperoxaluria, which may lead to the formation of calcium oxalate stones. Among 37 patients with idiopathic calcium oxalate stone, *Oxalobacter formigenes* in the intestinal microbiome was positive in 11 patients and negative in 26 patients. *Oxalobacter formigenes* was not detected in 70% of patients. In the case of multiple stone formers, it was found that 60–80% of patients were negative for *Oxalobacter formigenes*. However, there was no difference in the concentration of urine oxalate between positive and negative *Oxalobacter formigenes* in 24-h urinalysis. In the case of enteric oxaluria, 0.5 mmol/d or 40 mg/d or more should be detected, but hyperoxaluria was not seen at 0.3 and 0.4 mmol/d in both groups. Therefore, a contradiction occurred in the mechanism that the absence of *Oxalobacter formigenes*, which we already knew, induces hyperoxaluria. Ticinesi et al. [53] reported an urolithiasis and intestinal microbiome study that examined the cause of the contradiction between *Oxalobacter formigenes*, hyperoxaluria, and calcium oxalate stones. They analyzed the metagenomics of 52 stone formers and 48 healthy controls. However, very few *Oxalobacter formigenes* were found among 100 subjects, less than 0.001% of the total sample. Relative abundance also showed no difference. As a result of analyzing the concentration of oxalate in the 24-h urine test in both groups, the strains presented showed differences. Assimos commented regarding the results of this study [54], the abundance of *Faecalibacterium*, *Enterobacter* and *Dorea* reduced stone formation, and *Faecalibacterium* is recognized as generating short chain fatty acids. It is thought to attenuate the inflammation and oxidative stress associated with stone formation. The negative correlation between *Oxalobacter formigenes* and hyperoxaluria is because *Oxalobacter formigenes* was rarely found in the 100 subjects, and the results were different from those known so far. This is why, even in the absence of *Oxalobacter formigenes*, it does not cause enteric hyperoxaluria, showing the possibility that it is not a valid theory or that there is no clinical value in the theory. Bostanghadiri et al. [55] described that the contradiction of *Oxalobacter formigenes* could be due to a variety of factors, including the study’s population, lifestyle, and eating habits, all of which could affect the gut microbiome, particularly *Oxalobacter formigenes.*

Stern et al. [56] examined in a pilot study the significant variations in the gut microbiome of urolithiasis patients in comparison to patients without kidney stone formation. Their findings showed that the genus *Prevotella* was 2.8-fold more prevalent in the control group without kidney stones, but the genus *Bacteroides* was 3.4-fold more abundant in the kidney stone group. According to a 24-h urine examination, the genus *Eubacterium* was negatively correlated with oxalate levels and the genus *Escherichia* was negatively correlated with citrate levels. Recently, Kim et al. [57] conducted a prospective cohort study aimed at examining the association between the prevalence and incidence of renal stones and the gut microbiome in a relatively large-scale study of 915 participants. Patients were divided into the following groups according to the presence or absence of renal stones at the initial and subsequent visits: G0, no renal stones (control), those without renal stones at the initial and subsequent visits; G1, incidental renal stones, those without renal stones initially but with renal stones at the follow-up visits; and G2, prevalent renal stones, those with renal stones at the beginning of the experiment. The median follow-up period was 4.0 years (interquartile range, 2.0–5.0 years; maximum 5.5 years). The abundances of other taxa, as opposed to *Oxalobacter formigenes*, varied significantly between the control group and the renal stone group, as seen in prior reports, and their findings were consistent with the results of those studies. In contrast to the accidental stone group, they discovered that *Bifidobacterium* was more prevalent in the no stone group. *Dorea*, *Incertae sedis*, and *Faecalibacterium* abundances were discovered to be lower in the incidental stone group than in the no stone group. The incidental stone group also showed higher abundances of *Fusobacteria*, *Phascolarctobacterium*, and *Erysipelatoclostridium* and reduced abundances of *Eubacterium eligens group* and *Dialister* as compared to the no stone group. Finally, they observed *Faecalibacterium* was found in lower abundance in the incidental stone group than in the no stone group, but there was no difference in abundance between the prevalent stone and no stone groups. 

Deng et al. [58] reported that 16S ribosomal RNA (rRNA) gene sequencing reveals an altered composition of the gut microbiome in postoperative individuals with renal stones. They used 16S ribosomal RNA (rRNA) gene sequencing to examine the relationships between the gut microbiome and renal stone formation. In summary, 20 patients were chosen, and data on health and eating patterns from the previous 1–3 months were gathered at the time of admission. A total of 40 samples were examined, yielding 493 operational taxonomic units (OTUs), with an average of 67,888 ± 827 reads per sample. OTU-based partial least squares discriminant analysis (PLS-DA) analysis with OTU-based results revealed differences between the RS1 (fecal specimen taken before surgery) and RS2 (fecal specimen taken one month after surgery) groups, with a significantly greater level of OTU7 in the RS2 group. Taxonomy-based comparisons of the gut microbiome revealed changes in the flora composition, with greater prevalences of *Enterobacteriaceae*, *Gammaproteobacteria*, *Escherichia*, and *Enterobacteriales* in the RS2 group and *Pseudomonadaceae*, *Pseudomonadales*, and *Pseudomonas* in the RS1 group. According to correlation analysis, a lower level of urea was correlated with a higher prevalence of *Enterobacteriaceae*, *Gammaproteobacteria*, and *Escherichia*, while a lower level of creatinine was correlated with a higher frequency of *Escherichia*. These findings may offer new perspectives for the prevention, diagnosis, and treatment of renal stones as they strongly imply that the gut microbiome plays a significant role in kidney stone formation. 

Yuan et al. [59] reported an association of dietary patterns with gut microbiome in kidney stone and non-kidney stone individuals. In their study, both the calcium oxalate kidney stone group and the non-kidney stone group had a gut microbiome with significantly different abundances in the high urolithiasis risk dietary patterns compared to the low urolithiasis risk counterparts, while *Pseudomonas*, *Sphingomonas*, *Slackiain*, *Corynebacterium*, *Arcobacter*, *Stenotrophomonas, Hydrogenoanaerobacterium*, and *Faecalitalea* were found to be more abundant in the high urolithiasis risk kidney stone group, which is significant in the formation of kidney stones, so were metabolic pathways linked to inflammation, lipid, and mineral metabolism. Their results suggested that dietary habits may influence the prevention and treatment of calcium oxalate stones by controlling the gut microbiome’s homeostasis.

In a recent study by Xiang et al. [60], a machine learning model was developed to forecast the likelihood of calcium oxalate kidney stones based on a combination of clinical and gut microbiome features. The study included data from a total of 180 subjects, with 120 subjects allocated to the training set and the remaining 60 subjects used for validation purposes. The authors evaluated the performance of eight distinct machine-learning techniques using clinical and gut microbiome data from 66 non-kidney stone individuals and 54 kidney stone patients. They identified three stone-related bacteria (*Flavobacterium*, *Rhodobacter*, and *Gordonia*). Clinical data were comprised of five characteristics: oxalate concentration, acetic acid concentration, citrate concentration, phosphorus concentration, and urinary PH. Area under the curve (AUC) of predictive models using only three genus was 0.763, the AUC of using five pieces of clinical information was 0.902, and the AUC of using three genus plus five pieces of clinical information was 0.936. In the end, the gut microbiome plays a role in forming calcium oxalate stones, but it needs to be further elucidated in the future.

## 7. The Microbiome of Urinary Stones

The microbiome of urinary stones refers to the microbiome discovered using EQUC and 16S rRNA gene sequencing in stones themselves.

Bacteria can be identified in between 15 and 70 percent of stones [61,62,63]. In 13% to 44% of samples, calcium oxalate stones had positive cultures. The most frequent bacteria found in stone cultures were *E. coli* (15–35%), *Pseudomonas spp*., and *Proteus* (urease-producing bacteria), which are frequently linked to the development of struvite stones [62,63].

An analysis of the microbiome of calcium stones has recently been reported [15]. In 52 urolithiasis patients, patients who had used antibiotics within 4 weeks, pregnant or hospitalized patients, and patients with a history of struvite stones were excluded. After removing the stones with a ureteroscope and laser, an EQUC and 16S rRNA sequencing were performed. In 16s rRNA sequencing, 55.8% of cases were positive. After comparing with a negative control and removing difficult-to-identify stones, 12 stones were confirmed as sequencing positions, and 40 were considered negative. When the stones sequencing positive and negative were compared, calcium oxalate was confirmed in 90% of the negative group, and brushite was found in the positive group. Uric acid and cystine stones were found only in the negative group. The struvite stones were the same in both groups. When looking at the results of expanded culture and sequencing, various bacteria were distributed in the 12 stones, and very diverse bacteria were found in the sequencing metagenomics. The most prevalent bacterial taxon was *Enterobacteriaceae* (including the genera *Escherichia* and *Klebsiella)*, *Staphylococcus*, *Veillonella*, *Streptococcus*, *Corynebacterium*, *Haemophilus*, *Proteus*, *Lactobacilli*, and *Bifidobacterium*. Multiple enriched bacterial species were discovered using EQUC and 16S rRNA sequencing Bacterial species isolated from the urinary stone that were concordant with 16S rRNA gene sequencing identification include *S. epidermidis*, *E. cloacae*, *E. coli*, and *L. gasseri*. The authors describe the value of their research as follows: a method for comparing the microorganisms of urine and stone is presented, and it shows similar microbiomes of selective urine in the upper urinary tract and the bladder. A calcium oxalate stone indicates that the presence of bacteria is low. This result is inconsistent with the experiment mentioned above that showed *E. coli* in mice could promote calcium oxalate stone formation. In a meta-analysis, *Staphylococcus* and *Aerococcus* genera dominated the microbiome of stone samples across two studies, with *Enterobacteriaceae* present in high abundance based on OTUs, and *Enterococcus* predominant based on ASVs [14]. 

When there was a urinary tract infection, urolithiasis growth was observed in 61.5% of cases, and stones grew in only 12.5% of cases with no urinary tract infection [64]. This study demonstrates the need to treat urinary tract infection to prevent the progression of infectious stones. However, more research is needed to determine whether treating the microbiome in calcium oxalate stones as a prevention strategy is worthwhile.

## 8. Probiotics for Prevention of Urolithiasis

*Oxalobacter formigenes* may protect against calcium stones via two distinct mechanisms: oxalate degradation in the gut lumen, which reduces mucosal absorption, and promotion of endogenous oxalate secretion by the gut mucosa [65]. From *Eubacterium lentum*, the oxalate-degrading proteins oxalyl-CoA decarboxylase and formyl-CoA transferase was isolated [66,67]. Turroni et al. [68] discovered that *Bifidobacterium* subsp. *lactis* DSM 10140, *Bifidobacterium adolescentis* MB 238, and *Bifidobacterium longum* MB 282 had the highest levels of oxalate degradation. In addition, according to Turroni et al. [68], a variety of *Lactobacilli*. can degrade oxalate. The presence of *oxc* and *frc* genes was discovered in isolates of *Lactobacillus acidophilus* and *Lactobacillusgasseri* that degraded more than 50% oxalate. 

Wigner et al. [6] have reviewed the use of probiotics to prevent calcium oxalate stones. Prior research has mostly concentrated on administering *Oxalobacter formigenes* to patients with urolithiasis. Due to its antibiotic sensitivity and low pH, this bacterium is not a good probiotic. Thus, later research focused on well-known probiotics, including *Lactobacilli* and *Bifidobacterium* strains, *Eubacterium lentum*, *Enterococcus faecalis*, and *E. coli*, to identify bacteria that are capable of degrading oxalate. However, *Oxalobacter formigenes* has the best potential for oxalate degradation of all the bacteria examined. Subsequent research has revealed the existence of *Oxalobacter formigenes* strains that are resistant to low pH and contain oxygen, confirming the possibility of using *Oxalobacter formigenes* in clinical settings [69,70,71]. No reduction in the viable cell count was seen when the *Oxalobacter formigenes* strain was cultured in anaerobic media at pH 6.8 and pH 3.0 for up to 2 h [71]. Mass spectrometry (MS)-based shotgun proteomics study of anaerobic *Oxalobacter formigenes* cultures found superoxide dismutase (substantial protection against oxygen toxicity) to be expressed [70]. Additionally, it was demonstrated that several variables, including the acid produced by yogurt bacteria and oxygen permeability through the packaging, affect the survivability of probiotic bacteria in yogurt [72,73,74].

Hoppe et al. [69] conducted clinical study (16 patients with urolithiasis). There were two groups of patients. The first group consisted of nine patients who received *Oxalobacter formigenes* as a frozen cell paste containing 1 g of live cells equivalent to >10^10^ CFU (IxOC-2). The second group was made up of seven patients who received two enteric-coated capsules of *Oxalobacter formigenes* (137 mg of lyophilized bulk powder of freeze-dried live cells, equivalent to ~10^7^ CFU) per dose (IxOC-3). In order to evaluate the degree of oxalate extraction, urine and plasma samples were obtained. In their study, *Oxalobacter formigenes* intake can reduce the urinary oxalate levels in patients with urolithiasis (IxOC-2: 22–48%, IxOC-3: 38.5–92%). 

Combining probiotics is one possible solution to address the issue of antibiotic sensitivity. Such an approach may help mitigate the problem of intestinal colonization caused by antibiotic use. According to reports, combining different probiotics may also be a good solution because the entire intestinal microflora participates in the breakdown of oxalate and reduces its excretion in urine [75,76]. Campieri et al. [76] discovered that a daily dose of a mixture of freeze-dried *Lactobacilli* strains (*L. acidophilus, L. plantarum, and L. brevis*), *Bifidobacterium infantis*, and *Streptococcus thermophilus* (administered as a daily dose at 8 × 10^11^ CFU, 4 weeks probiotic therapy) caused a significant reduction in urinary oxalate excretion, i.e., by about 40%, in six patients with idiopathic calcium oxalate urolithiasis and mild hyperoxaluria. Wigner et al. [6] concluded that it is necessary to perform additional research with a bigger population under highly controlled circumstances. No studies have attempted to comprehensively evaluate changes in the gut microflora, and most studies rely on small study groups, which frequently follow an unplanned diet. Additionally, there are discrepancies between the ability of these bacteria to metabolize oxalate in vitro and in vivo, according to studies on *Lactobacilli* and *Bifidobacterium* strains, *E. lentum*, *E. faecalis*, and *E. coli* that have primarily been conducted in animals and in vitro [7,66,67,68,75,76,77,78,79,80,81,82,83,84,85,86,87,88,89,90,91,92]. 

Stepanova et al. [10] have performed experiments (i) to evaluate whether ceftriaxone treatment could affect the number of intestinal oxalate-degrading bacteria, their overall oxalate degradation activity, and their influence on oxalate homeostasis in rats, (ii) to assess the impact of commercially available probiotics and synbiotics (food ingredients or dietary supplements combining probiotics) on total fecal oxalate-degrading activity, and (iii) to measure how well synbiotics can reverse ceftriaxone-induced disturbance of the oxalate homeostasis and fecal oxalate-degrading activities in rats. They randomly divided 28 female Wistar rats (weighing 200–300 g) into four groups (*n* = 7). Group 1 (control) was treated with sterile water (0.1 mL, i.m., 14 days); Group 2 was treated with synbiotics (30 mg/kg, per os, 14 days); Group 3 was treated with ceftriaxone (300 mg/kg, i.m., 7 days); and Group 4 was treated with ceftriaxone and synbiotic. On days 1 and 57 following the termination of treatment, the number and total activity of oxalate-degrading bacteria, as well as urine and plasma oxalate concentrations, were assessed. Oxalate homeostasis was altered by ceftriaxone treatment, which also significantly increased urine oxalate levels. The number of oxalate-degrading bacteria in the fecal microbiome did not differ significantly between the groups on day 57 following treatment discontinuation. However, total oxalate-degrading activity was significantly higher in both synbiotic treatment groups than in the control and ceftriaxone alone-treated groups. Urinary oxalate excretion was markedly decreased in the rats given synbiotics. The overall amount of oxalate-degrading activity in the fecal microbiome was not correlated with the number of oxalate-degrading bacteria, and this activity was inversely correlated with plasma and urine oxalate concentrations. These findings imply that supplementation with synbiotics enhances the total oxalate-degrading capacity of the gut microbiome, leading to a substantial reduction in urine oxalate excretion [9]. Ticinesi et al. [78] reviewed the effects of the administration of oxalate-degrading bacteria on lithogenic risk. Overall, the findings were inconsistent, with some studies reporting significant decreases in urinary oxalate excretion following probiotic treatment [71,76,80,84,86,91], while others showed no changes from baseline [81,89]. Regarding the inconsistency of findings, the authors noted that oxalate excretion represents a proxy endpoint for stone recurrence and is merely one of several factors that contribute to the definition of lithogenic risk [78].

## 9. Conclusions

The urinary microbiome could affect calcium oxalate supersaturation, biofilm formation and aggregation, and urothelial injury. Standardization (study design, type of sample, sample collection, sample storage, DNA extraction methods, and data analysis) is needed in urine microbiome research for urolithiasis. 

## Figures and Tables

**Table 1:** Urinary microbiome associated with urolithiasis.

Urinary Microbiome (Urinary Stone Disease Group)	Urinary Microbiome (Healthy Group)
*Escherichia coli*	*Lactobacilli*
*Staphylococcus aureus*	*Bifidobacterium*
*Streptococcus pneumoniae*	*Veillonellaceae*
*Pseudomonas aeruginosa*	
*Burkholderia*	
**Urease-producing Organisms**	
*Proteus mirabilis*	
*Klebsiella pneumonia*	
*Staphylococcus aureus*	
*Pseudomonas aeruginosa*	
*Providencia stuartii*	
*Serratia marcescens*	
*Morganella morganii*	

## Data Availability

Not applicable.
